# Biotin-Conjugated Cellulose Nanofibers Prepared via Copper-Catalyzed Alkyne-Azide Cycloaddition (CuAAC) “Click” Chemistry

**DOI:** 10.3390/nano10061172

**Published:** 2020-06-16

**Authors:** Katarina Goodge, Margaret Frey

**Affiliations:** Department of Fiber Science and Apparel Design, Cornell University, Ithaca, NY 14853, USA; keg222@cornell.edu

**Keywords:** electrospin, nanofiber, cellulose, CuAAC, click, biotin, selective capture

## Abstract

As potential high surface area for selective capture in diagnostic or filtration devices, biotin-cellulose nanofiber membranes were fabricated to demonstrate the potential for specific and bio-orthogonal attachment of biomolecules onto nanofiber surfaces. Cellulose acetate was electrospun and substituted with alkyne groups in either a one- or two-step process. The alkyne reaction, confirmed by FTIR and Raman spectroscopy, was dependent on solvent ratio, time, and temperature. The two-step process maximized alkyne substitution in 10/90 volume per volume ratio (v/v) water to isopropanol at 50 °C after 6 h compared to the one-step process in 80/20 (v/v) at 50 °C after 48 h. Azide-biotin conjugate “clicked” with the alkyne-cellulose via copper-catalyzed alkyne-azide cycloaddition (CuAAC). The biotin-cellulose membranes, characterized by FTIR, SEM, Energy Dispersive X-ray spectroscopy (EDX), and XPS, were used in proof-of-concept assays (HABA (4′-hydroxyazobenzene-2-carboxylic acid) colorimetric assay and fluorescently tagged streptavidin assay) where streptavidin selectively bound to the pendant biotin. The click reaction was specific to alkyne-azide coupling and dependent on pH, ratio of ascorbic acid to copper sulfate, and time. Copper (II) reduction to copper (I) was successful without ascorbic acid, increasing the viability of the click conjugation with biomolecules. The surface-available biotin was dependent on storage medium and time: Decreasing with immersion in water and increasing with storage in air.

## 1. Introduction

Current biosensor research tackles performance issues by immobilizing probes on planar surfaces in point-of-care devices. However, the largest drawback is that the poor surface area limits the attachment of probes, directly correlating to lower sensitivity and higher response time [1]. Instead, 3D structures are being explored for their high surface-area-to-volume ratio, enhanced roughness, and porosity. Increased reproducibility is gained from the consequent higher probe loading. Fabrication methods include photolithography, metal etching, and layer-by-layer growth of nanotubes/particles, but these are expensive, time consuming, and process intensive. Furthermore, the probe immobilization techniques have added drawbacks including probe leaching, deactivation of the probe during immobilization, low loading capacity, and poor shelf life [2,3,4,5].

Nanofibers are an attractive option due to their ease of formation, high surface-area-to-volume ratio, and range of potential functionalization. The high surface-area-to-volume ratio corresponds to high availability of active sites on the surface and is crucial in extending the sensitivity of the resulting assay. Out of the multitude of techniques to produce nanofibers, including drawing, template synthesis, phase separation, self-assembly, and electrospinning, electrospinning is the only process that has successfully fabricated nanofiber membranes on an industrial scale [6]. Multi-jet, needle-free spinnerets and roll-to-roll collector systems such as Nanospider™, Inovenso, and Bioinicia mitigate the lab-scale drawbacks of low throughput, and adaptable configurations of the equipment make electrospinning an attractive option for a wide range of applications. Useful overviews of the fundamentals of nanofiber formation via electrospinning and applications of electrospun fibers have been published [6,7,8,9,10]. Cellulose acetate was chosen for this study based on its low cost from a renewable source, spinnability, and ability to readily functionalize with various chemistries. The cellulose acetate nanofibers can be functionalized via their acetyl groups, or they can be deacetylated to take advantage of the change in reactivity of the hydroxyl group on cellulose carbon-6 [10]. 

The simplest technique for immobilizing molecules onto substrate surfaces is physical immobilization such as adsorption and entrapment. These are not sufficient for applications requiring that the immobilized biomolecules maintain functionality and remain on the substrate surface without leaching during washing or storage [11]. These challenges can be overcome by permanently attaching the biomolecules to the substrate with covalent linkages. Some strategies for biomolecule attachment to nanofibers include N-ethyl-N′-(3-(dimethylamino)propyl) carbodiimide/N-hydroxysuccinimide (EDC/NHS) coupling [2], crosslinking with glutaraldehyde (GA) vapor [3], and creating reactive sites with plasma [4]. While the coupling chemistries mentioned above have varying degrees of success in short-term stability, reusability, and bioactivity, these methods are susceptible to hydrolysis and degradation with long-term storage [12]. As anticipated applications of the functionalized nanofibers will be in aqueous systems, such as filtration, microanalytical devices, catalysts, etc., water instability is unfavorable.

A relatively new post-electrospinning modification strategy, click chemistry, allows for site-specific bioconjugation. Click chemistry is a class of specific reactions, characterized by relatively fast, catalyzed reactions, high efficiency, high selectivity, and stable reactants, that was originally named by Karl Barry Sharpless in 2001 [13]. A key advantage of click reactions is that they are bio-orthogonal, and, therefore, biomolecules can participate in the reactions while preserving their bioactivity [13]. The specific click reaction used in this work, copper-catalyzed azide-alkyne cycloaddition (CuAAC), is irreversible and highly selective to only couple azides and alkynes to form a triazole ring [14,15,16,17]. The alkyne attachment reaction to the nanofiber and the click reaction between the alkyne and azide were both achieved under mild conditions, with the latter performed in an aqueous solution at room temperature. Click modification of polysaccharides has been widely studied, yet central reviews on this topic do not discuss cellulose nanofibers as click substrates [18,19]. Further, various click reactions have been studied as bioconjugation techniques with nanofibers [20,21,22], but only a few recent studies have reported CuAAC clicking with cellulose-based fibers [23,24,25]. These studies employed the click reactions in nanofiber systems but did not further investigate the dynamics of the heterogeneous reaction on the porous, sorbent nanofiber membranes.

Herein, cellulose nanofibers were spun via electrospinning, modified with propargyl bromide to create reactive alkyne sites, and clicked with azide-biotin conjugate. The biotin yield as a function of intermediate functionalization and click reaction conditions was characterized. The click reaction was studied by varying the ratio of ascorbic acid to copper sulfate as well as the various permutations of the click reaction components to elucidate the interactions of each component with the cellulose nanofiber membranes. The biotin-cellulose fibrous membrane was used to specifically and rapidly detect streptavidin present in the fluid sample based on the biotin-streptavidin binding affinity. By covalently bonding biorecognition molecules to the nanofiber surface, these biotin-cellulose membranes demonstrate the potential for immobilizing specific antibodies to the surface of the nanofibers to be used in a portable, nonwoven diagnostic device.

## 2. Materials and Methods 

### 2.1. Materials

Cellulose acetate (CA) (average molecular weight (Mn) = 30,000, acetyl content = 39.8%) was purchased from Sigma-Aldrich, St. Louis, MO, USA. Dichloromethane (DCM) (Fisher Chemical, Hampton, NH, USA), isopropanol (IPA) (Fisher Chemical), methanol (Fisher Chemical), ethanol (Decon Labs, King of Prussia, PA, USA), potassium hydroxide (KOH) (Mallinckrodt, St. Louis, MO, USA), sodium hydroxide (NaOH) (Avantor, Radnor Township, PA, USA), propargyl bromide (PBr) (Sigma-Aldrich), L-ascorbic acid (Sigma-Aldrich), copper (II) sulfate anhydrous (Sigma-Aldrich), azide-(poly(ethylene glycol))biotin (azide-PEG3-biotin) conjugate (Sigma-Aldrich), streptavidin-fluorescein-isothiocyanate (FITC) from Streptomyces avidinii (Millipore Sigma, Burlington, MA, USA), and PierceTM Biotin Quantification Kit (Thermo Scientific, Waltham, MA, USA) were used without further purification.

### 2.2. Electrospinning

CA electrospinning solution was prepared at 12% weight per volume ratio (w/v) polymer concentration in 4/1 volume per volume ratio (v/v) DCM/methanol solvent mixture. The solution was added to a 5-mL BD plastic syringe (inner diameter = 11.989 mm) with BD PrecisionGlide needle (21 gauge (G), 50-mm length). A high voltage supply (Gamma high Voltage Research) provided 15 kV to the needle tip and a grounded copper collector was placed 12 cm away. The solution feed rate was maintained at 1.0 mL/h using a programmable PHS Ultrasyringe pump (Harvard Apparatus). Electrospinning took place in a Plexiglas humidity-control box (Plas Labs) at 20–60% relative humidity and room temperature. The resulting nonwoven membrane was kept in 40 °C oven overnight to remove residual solvent.

### 2.3. Regeneration of Cellulose (Scheme 1a)

**Scheme 1:**
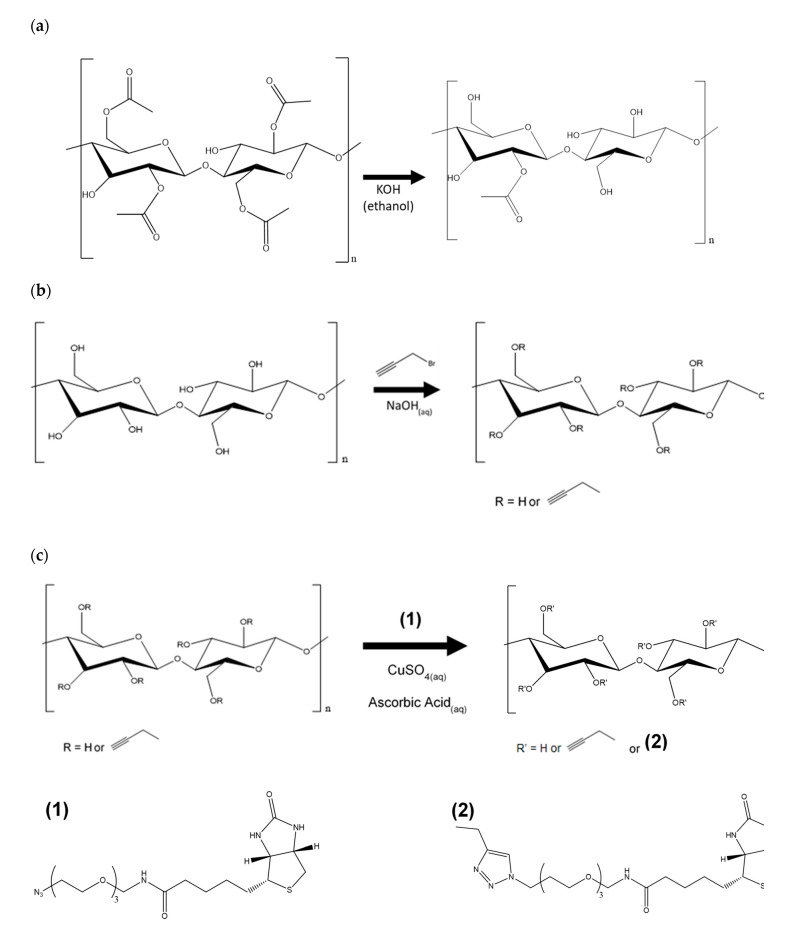
Reaction scheme for (**a**) regeneration of cellulose, (**b**) alkyne substitution, and (**c**) Copper-catalyzed Alkyne-Azide Cycloaddition (CuAAC) click.

In Scheme 1a, deacetylation of the CA nanofibers was achieved with 0.5 Normal (N) KOH in ethanol solution at constant shaking at room temperature [26]. After 3 h, the regenerated cellulose (RC) membrane was rinsed with deionized water until pH paper confirmed neutral pH and placed in oven at 40 °C overnight. 

### 2.4. Alkyne Substitution (Scheme 1b)

Scheme 1b is adapted from Mangiante [27]. Briefly, nanofiber membranes were added to IPA (12.75 mL/mmol CA, 48.5 mL/mmol RC) in a flask and let sit for 15 min. Then, 5.1 wt.% NaOH (aqueous) (40.0 mL/mmol CA, 4.25 mL/mmol RC) was added to the reaction mixture in 0.5-mL increments over 15 min to avoid chemically destroying the nanofibers. The reaction mixture was heated over one hour while shaking, and propargyl bromide (4.5 mL/mmol fiber) was added via syringe. Temperature and shaking were maintained for specified time. For each sample removal, the flask was removed from water bath and let cool for 30 min. The nanofiber mat was rinsed with water, then IPA, then IPA/water mix, then it sat in water for 10 min to ensure all unreacted materials had diffused from the mat. Lastly, the mat was dried in 40 °C oven overnight.

### 2.5. Click Reaction with Azide-Biotin Conjugate (Scheme 1c)

Scheme 1c is adapted from Celebioglu [25]. Briefly, the azide-biotin conjugate (1 equiv) was dissolved in 33.3 mL water per mmol fiber, and the nanofiber membrane was added to the solution. Fresh solutions of ascorbic acid (2.5 equiv, clear solution) and copper sulfate anhydrous (2.5 equiv, light blue solution) were prepared. The copper sulfate solution was added to the azide-biotin conjugate solution first, then the ascorbic acid solution was added. The mixture was shaken at room temperature. After the specified time, the nanofiber membrane was removed from the solution, rinsed with water, and let soak in fresh water for three days to ensure all unreacted biotin diffused from the membrane. The biotin-cellulose membrane was rinsed briefly with water and dried in 30 °C oven overnight.

### 2.6. Characterization

Fiber morphology and diameter were examined using scanning electron microscopy (Zeiss Gemini 500 SEM) with an accelerating voltage of 2 kV. ImageJ™ open source software (National Institutes of Health) was used to measure the mean fiber diameters. Fifty measurements were taken for each sample from three separate images. Energy Dispersive X-ray spectroscopy (EDX) spectra were collected with an accelerating voltage of 10 kV. Samples were sputter coated with carbon for SEM and EDX. Chemical structure was analyzed with Attenuated Total Reflection-Fourier Transform Infrared (ATR-FTIR) spectroscopy (ATR-FTIR Spectrometer PerkinElmer Frontier) at each reaction step. Each spectrum was an average of 128 scans obtained between 4000 and 600 cm^−1^ at a resolution of 4 cm^−1^. Four spectra were collected per sample. Alkyne substitution was confirmed with Raman spectroscopy (Renishaw InVia Confocal Raman microscope). Each spectrum was obtained between 3250 and 200 cm^−1^ with an excitation wavelength of 785 nm and a resolution of ~1 cm^−1^. Three spectra were collected per sample. Crystalline structure was analyzed with X-ray diffraction (Bruker D8 Advance ECO powder diffractometer, 1 kW Cu-Kα source). Three spectra were collected per sample. 

### 2.7. Streptavidin Binding and Confocal Microscopy

Static binding of streptavidin-FITC was carried out in microwell plate; 4 mm × 4 mm fiber samples were wet with Phosphate-Buffered Saline (PBS) solution (0.300 mL) in microwell plate overnight then let dry. Then, 100 μL of 1 micromolar (μM) streptavidin-FITC solution per 0.70 mg of fiber was added to each well. Fiber membranes soaked for 1 h. Each sample was rinsed with PBS solution 3 times then placed in transportation containers to bring to confocal microscope. Bound streptavidin on the nanofiber surface was imaged using Zeiss LSM710 confocal microscope with water immersed 40× objective and 488 laser line. Emission and detection wavelength ranges were 488–563.5 nm and 493–634 nm, respectively. 

### 2.8. Surface-Available Biotin Quantification

The surface-available biotin was quantified using a competitive colorimetric assay kit. The absorbance was measured at 500 nm using a Lambda 35 UV/Vis Spectrophotometer from Perkin Elmer. The HABA (4′-hydroxyazobenzene-2-carboxylic acid)/avidin solution was reconstituted in 100 μL of ultra-pure water, added to 1000 μL of 1× PBS buffer solution, and the absorbance was measured. A preweighed piece of fiber mat was placed in the cuvette and shaken for 3 min. The absorbance was measured with and without the fiber mat. The surface-available biotin was calculated using the following equation:

*Surface-Available Biotin (mg biotin/g fiber) = (A^0^_500_−A_500_) (MW_biotin_V/εbW) × 10^3^*, (1)

where *A^0^_500_* is the absorbance of the solution prior to the addition of nanofiber mat, *A_500_* is the absorbance of the solution after reaction with nanofiber mat. *MW_biotin_* is the molecular weight of the biotin (244.3 g/mol), V is the volume of the solution (L), b is the cuvette path length (1 cm), *ε* is the extinction coefficient of the HABA/avidin complex at 500 nm (3.4 × 103 L/(mol cm)), and W is weight of the surface shell of the fiber (g). The surface-available biotin was used to calculate the degree of substitution of biotin (*DS_HABA_*) on the cellulose nanofibers:

*DS_HABA_ = [Surface-Available Biotin] × MW_RC_/(10^3^ × MW_biotin_)*, (2)

where *MW_RC_* is the molecular weight of one anhydrous glucose unit (AGU) (162.14 g/mol).

Samples were also analyzed with X-ray photoelectron spectroscopy (XPS) using a Scienta Omicron ESCA-2SR with operating pressure ca. 1 × 10^−9^ Torr. Monochromatic Al Kα X-rays (1486.6 electronvolt (eV)) with photoelectrons collected from a 2-mm diameter analysis spot. Photoelectrons were collected at a 0° emission angle with source-to-analyzer angle of 54.7°. A hemispherical analyzer determined electron kinetic energy, using a pass energy of 200 eV for wide/survey scans, and 50 eV for high resolution scans. A flood gun was used for charge neutralization of non-conductive samples. Degree of substitution of azide-PEG3-biotin conjugate onto alkyne-RC nanofibers was calculated based on the ratio of sulfur to carbon obtained from the XPS scans:

*DS_XPS_ = (72.06 × (S/C))/(32 − (12 × (S/C)))*. (3)

## 3. Results

### 3.1. Morphological Characterization 

**Figure 1:**
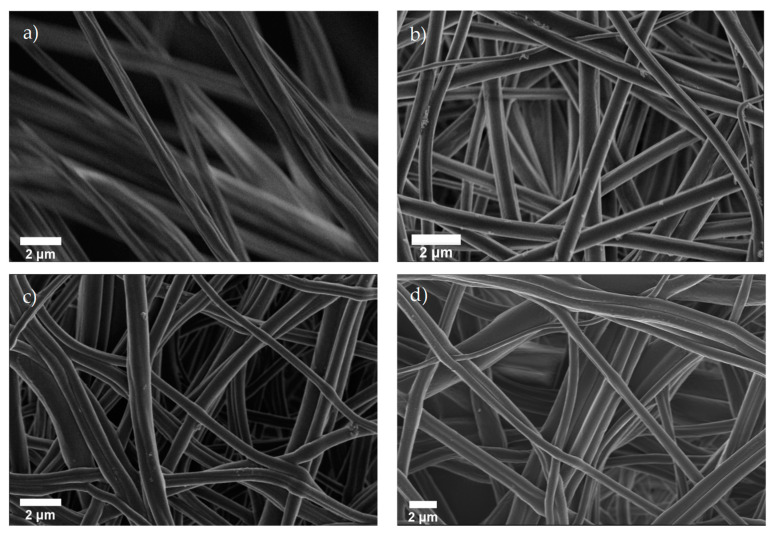
SEM images of (**a**) as-spun cellulose acetate (CA), (**b**) regenerated cellulose (RC), (**c**) alkyne-cellulose, and (**d**) biotin-cellulose nanofibers.

Cellulose acetate (CA) was electrospun into fibrous, nonwoven membranes then deacetylated to regenerated cellulose (RC), grafted with alkyne moiety (alkyne-cellulose), and finally clicked with azide-biotin conjugate (biotin-cellulose). SEM images of the nanofiber membranes at each reaction step are compared in Figure 1. The rough surface of the cylindrical as-spun fibers (Figure 1a) became smooth and round after deacetylation (Figure 1b). Swelling of the cellulose fibers during the alkyne substitution and click reaction steps caused the irregular appearance observed in Figure 1c,d, respectively. Figure 1c depicts the two-step alkyne substitution sample but is representative of both the one- and two-step alkyne substitution processes; neither process negatively impacted the fiber morphology at the respective optimal reaction conditions. Figure 1d illustrates a successful click reaction of the 10:1 AA:Cu ratio for 48 h and is representative for the click samples listed in Table 1. 

Temporary needle blockage caused side jets to form that reduced the diameter of the fibers and resulted in a skewed fiber diameter distribution (Figure S1a) with a mean of 741 nm and median of 680 nm. The diameter of CA fibers from as-spun to deacetylated (Figure S1b) was not significantly reduced, but the alkyne-cellulose fiber diameters (Figure S1c) were significantly reduced relative to both the as-spun and deacetylated CA fibers. The change in hydrogen bonding, removal of the acetyl groups, and addition of alkyne groups contributed to the changes in fiber diameter. No trend was found between successful click reactions and significant change in fiber diameter (Figure S2). Although no definite size was provided by the supplier, the azide-biotin conjugate had a molecular weight of 444.55 g/mol and was distinctly smaller than the diameter of the nanofibers. Therefore, the biotin addition was not the only factor in the change of fiber diameter. 

### 3.2. Alkyne Substitution

CA nanofibers were modified to obtain alkyne-cellulose nanofibers, either in a one- or two-step process. In the one-step process, CA nanofibers were deacetylated and alkyne substituted in one pot. In the two-step process, CA nanofibers were first deacetylated to regenerated cellulose (RC) nanofibers, and the alkyne groups were attached to the RC nanofibers in a separate step. The nucleophilic substitution of the alkyne group onto the nanofiber surface was tested at three levels of temperature and six time intervals for both CA (one-step) and RC (two-step).

**Figure 2:**
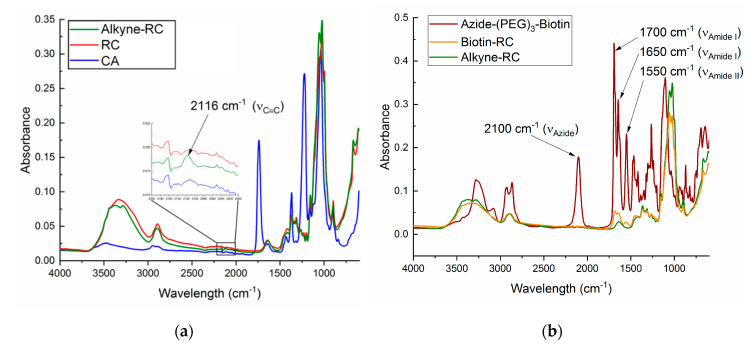
FTIR spectra of (**a**) as-spun CA, RC, and alkyne-RC nanofibers and (**b**) alkyne-RC, biotin-RC, and azide-(poly(ethylene glycol))biotin (azide-PEG3-biotin) conjugate.

ATR-FTIR was used to monitor the changes in sample surface chemistry. Representative spectra for each step are shown in Figure 2a. The characteristic absorption bands for CA appeared at 1740, 1226, and 1368 cm^−1^, attributed to the C=0 and C-O stretching and C-H bending vibration of CH_3_ of the acetyl group, respectively. The broad bands at 2850–2950 and 3400–3500 cm^−1^ were attributed to the C-H stretching of CH_2_ or CH_3_ and -OH stretching of unacetylated cellulose, respectively. The RC spectrum showed changes in these characteristic peaks to resemble native cellulose. Specifically, the disappearance of the 1740, 1226, and 1368 cm^−1^ peaks and increase in the 3100–3500 cm^−1^ band confirmed the removal of the acetyl groups and exposure of the hydroxyl groups. 

**Figure 3:**
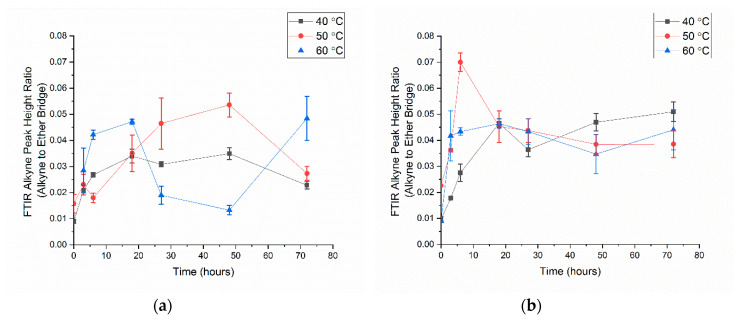

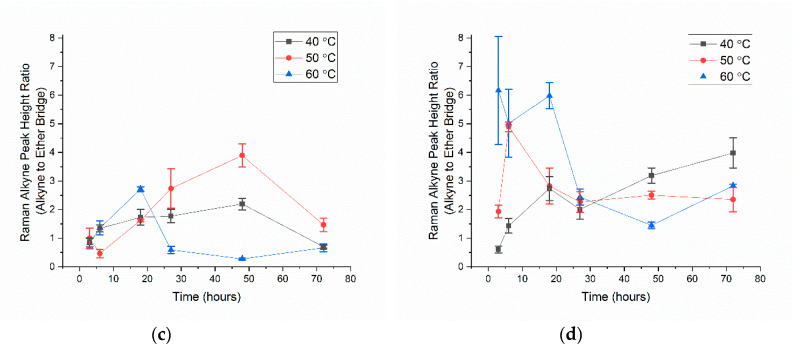
Alkyne substitution reaction yield as a function of temperature and time compared using (**a**,**b**) FTIR and (**c**,**d**) Raman spectra’s alkyne peak height ratios for (**a**,**c**) one-step and (**b**,**d**) two-step RC nanofibers.

The alkyne peak at 2116 cm^−1^ was small for FTIR, even at high concentrations of the alkyne group (see inset of Figure 2a). So, for low concentrations, it was difficult to differentiate the alkyne substitution from the background noise. However, Raman spectroscopy showed an intense, sharp peak for the alkyne group at 2100 cm^−1^ (Figure S3). So, coupled with the low noise-to-signal ratio, Raman is a more reliable way to show that a sample contains an alkyne group. Raman is difficult to perform on nanofibers due to their curvature and, therefore, both FTIR and Raman spectroscopy were employed for the alkyne substitution. Care was taken to obtain reproducible spectra. Changes in the sharpness of the broad band between 3100 and 3600 cm^−1^ confirmed the change in the bonding of the hydroxyl groups and disruption of the hydrogen bonding. The ratio of the peak height of the alkyne group at 2115 cm^−1^ (FTIR) and 2100 cm^−1^ (Raman) to the reference peak of the ether bridge at ~1155 cm^−1^ was calculated to correlate the degree of alkyne substitution to reaction condition (Figure 3).

For the one-step trials, the carbonyl peak of the acetyl group disappeared in the FTIR spectra within the first 3 h. The calculated peak height ratios were relative but showed trends in the alkyne substitution. The FTIR and Raman data followed the same trends for all trials except for the RC at 60°C. This trial exhibited a high peak height ratio and high standard deviation for the 3-h Raman, while all other trials started with a low substitution at 3 h and increased over time before reaching a maximum and subsequently decreasing the peak height ratio. The alkyne substitution reached a maximum peak height ratio for each temperature due to the competition between the nucleophilic substitution of the alkyne group, side reactions, and degradation of the nanofiber mat. The Raman showed that as the reaction progressed (3 to 18 h), the standard deviation decreased, and the fibers became more uniformly substituted. After 18 h, hydrolysis of the glucose units resulted in loss of the alkyne substitution and a decrease in the alkyne peak ratio. 

FTIR and XRD were used to monitor the change in crystallinity after the deacetylation and alkyne substitution reactions. Amorphous as-spun CA fibers became more ordered upon removal of the acetyl groups and rearrangement of the inter- and intra-molecular hydrogen bonds to form the stable RC nanofibers. Increasing the deacetylation time led to increase in chain orientation. For the alkyne substitution, any order obtained during deacetylation was lost with increase in reaction time until an equilibrium was reached for the two-step RC (Figure S4a). The amorphous as-spun CA in the one-step process transitioned into regenerated cellulose as the reaction proceeded and also reached an equilibrium (Figure S4b). Therefore, the alkyne substitution reaction only took place at the surface, and the bulk of the fiber was unaffected.

RC membranes maintained sufficient strength and dimensional stability to process further in the click reaction step. The CA membranes, however, were weak and did not retain sufficient strength for further handling. Regenerating cellulose nanofibers and separately substituting the alkyne group was a milder process that reduced the overall reaction time from 48 h to 8 h. Optimal conditions chosen for alkyne substitution were RC in 20/80 (v/v) IPA/water at 50 °C for 6 h. Samples prepared under these conditions were used for the click reaction investigation of biotin immobilization.

### 3.3. Biotin Immobilization

Alkyne-substituted RC mats and azide-biotin conjugate participated in the copper(I)-catalyzed alkyne-azide cycloaddition (CuAAC) click reaction. Copper (II) sulfate was used as the source of copper, and ascorbic acid acted as a reducing agent to reduce copper (II) to copper (I). The copper (I) subsequently catalyzed the click coupling of the alkyne attached to the nanofiber mat and the azide attached to the biotin conjugate to form the triazole ring that linked the biotin to the cellulose nanofibers. Then, 2, 5, and 10 equiv of ascorbic acid to copper sulfate were tested alongside six controls in the following permutations, listed in Table 1, to elucidate the interactions of each component with the RC nanofiber membranes.

FTIR was used as first indication of successful biotin immobilization (Figure 2b). The characteristic absorption bands for the biotin conjugate appeared around 1700, 1650, and 1550 cm^−1^, due to amide I and II of the carbamide of biotin and amide of the peptide linkage of biotin to PEG section of the conjugate. Amide I band was due to C=O stretching vibrations of the peptide bonds whereas the amide II band was due to C-N stretching vibrations in combination with N-H bending. The azide group in the biotin conjugate at 2100 cm^−1^ disappeared after the click reaction, but the 1700, 1650, and 1550 cm^−1^ peaks remained in the successfully clicked samples. All samples with full reaction components and Control 5 (Table 1) gained biotin peaks, whereas the remaining control samples did not show evidence of biotin (Figure S5). 

Elemental mapping was used to analyze the distribution of biotin (Figure S6). Nitrogen, only present in the biotin conjugate, was seen along the fibers and not in the interstitial spaces. This confirmed that the biotin conjugate was not simply just entangled in the pores of the membrane but immobilized on the surface of the nanofibers. The nitrogen along the fibers was consistently seen in all the same trials as the ones with biotin FTIR peaks. No nitrogen was detected for the remaining samples.

**Figure 4:**
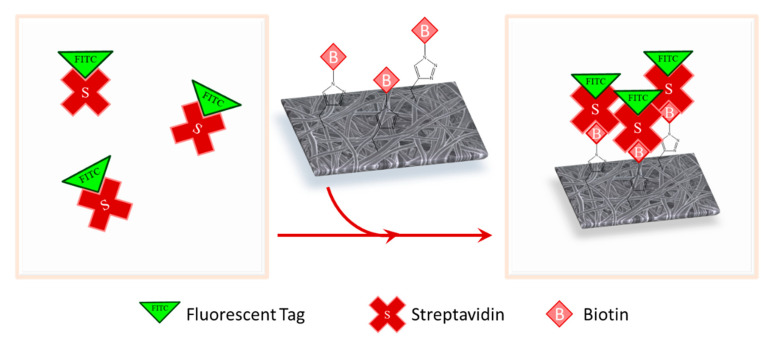
Streptavidin-biotin binding schematic: Fluorescently tagged streptavidin in aqueous solution binds with the immobilized biotin on the nanofiber surface once the membrane is added to the solution.

For the biotin-cellulose nanofiber membranes to be used in a diagnostic device, the membranes must be able to selectively and rapidly bind streptavidin. Fluorescently tagged streptavidin (streptavidin-FITC) was used as the model binding molecule. The binding schematic is shown in Figure 4. 

Confocal microscopy was used to image the fluorescent emission of the bound streptavidin-FITC (Table 1). Z stack images were taken to ensure the fluorescence or lack thereof was throughout the membrane and not solely on the surface of the membranes. The lack of fluorescence of Control 3 confirmed that no nonspecific binding of the streptavidin-FITC occurred when biotin was absent. Control 1 and 2 showed that the alkyne moiety was required for the biotin conjugate to be immobilized and bind with the streptavidin-FITC; the biotin conjugate did not physically absorb onto the fibers. Control 5 contained copper sulfate but not ascorbic acid to reduce the copper (II) to its catalyst copper (I) oxidation state, yet successfully bound some biotin conjugate. Control 6 contained no catalyst and could only react at uncatalyzed amounts. Therefore, both Control 5 and 6 had less intense fluorescence than the full reaction sample due to smaller initial yield. 

**Figure 5:**
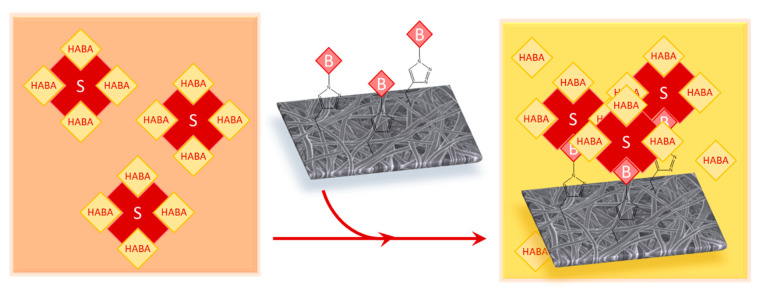
HABA (4′-hydroxyazobenzene-2-carboxylic acid) colorimetric assay: Streptavidin forms a complex with HABA molecules initially. Once biotinylated membrane is added to the mixture, the streptavidin will favorably bind with the available biotin. The change in absorbance at 500 nm correlates to the surface-available biotin.

The click reaction yield was quantified using HABA colorimetric assay and XPS. Figure 5 describes the HABA assay process for the biotin-cellulose nanofiber membrane samples. Each sample was given sufficient time to allow for the streptavidin-HABA complex to diffuse through the porous membrane and reach all available biotin molecules bound to the surface of the nanofibers. 

**Figure 6:**
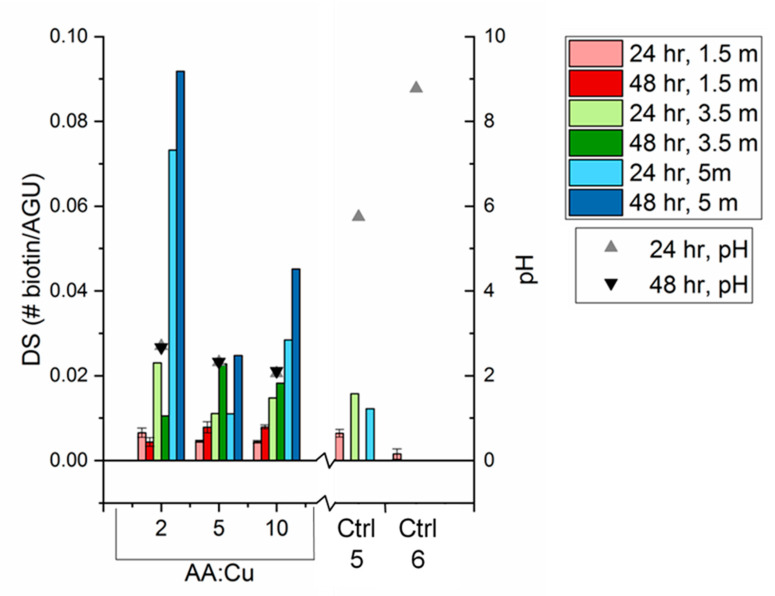
Degree of substitution of biotin on cellulose nanofibers, calculated from HABA colorimetric assay, increases with pH of reaction conditions and aging over a period of 5 months. All samples were stored in air for 5 months, and portions of each sample were assayed at 1.5, 3.5, and 5 months. Full reaction samples were tested at 2, 5, and 10 ascorbic acid-to-copper ratio (AA:Cu) for 24 and 48 h reaction times. All controls were quantified, and only Controls 5 and 6, shown here, had detectable biotin.

Surface-available biotin and degree of substitution were calculated using Equations (1) and (2), respectively. The HABA colorimetric assay was performed on the biotin-cellulose samples at 1.5, 3.5, and 5 months of aging in a desiccator, shown in Figure 6. The 1.5-month samples were measured on four separate pieces of the original mat. The 3.5- and 5-month samples were only measured on one piece of original mat each. Controls 1–4 had no detected biotin and were omitted from Figure 6. Control 6 had no detected biotin for the 3.5- and 5-month samples but is included in Figure 6 to show the significance of the reaction solution’s pH. 

The HABA assays were first conducted 1.5 months after the reactions were completed and showed that, within the 1.5-month sample set, the biotin yield increased with both increase in reaction time and ascorbic acid-to-copper sulfate ratio. The excess ascorbic acid ensured that enough Cu(II) was reduced to Cu(I) so as to not be the limiting step in the reaction. The ascorbic acid also lowered the pH of the solution, below the isoelectric point (pI) of the biotin (5.1), and can also denature proteins. At low pH, the protonated biotin had reduced its reactivity. Control 5 reaction did not contain ascorbic acid and ran at a pH above biotin’s pI. The measured biotin was higher than any of the trials with both components of the catalyst. The copper (I) was previously proven to be generated via alcohol oxidation and terminal alkyne homocoupling [28]. Removing the need for a reducing agent will be important for bioconjugation applications, such as direct click of proteins or antibodies on the nanofibers, since ascorbic acid reduces the pH into the range that can disrupt the structure of biomolecules. The reduced form of ascorbic acid, dehydroascorbate, can react with certain amino acids that change the protein functionality and/or cause protein aggregation [28]. Control 6 had no ascorbic acid or copper sulfate, so only a negligible amount of uncatalyzed click reaction was occurring. Control 6 further supported the assertion that Control 5 was being catalyzed via an alternative copper-reducing route. 

All click reaction samples were stored in a desiccator for 5 months and were retested at the 3.5- and 5-month marks. Overall, surface-available biotin increased exponentially with storage time for all full-reaction samples. Control 5 maintained a more stable amount of surface-available biotin. Both alkyne and biotin have lower surface energy than cellulose, so they should stay on the surface and not migrate to the fiber interior. Since the click reaction was carried out in aqueous solution, the water molecules disrupted the intra- and inter-molecular hydrogen bonding of the cellulose chains, causing the cellulose fibers to swell in the aqueous solution. Once the cellulose membrane was removed from the aqueous solution, the hydroxyl groups remained in their conformation, which inhibited biotin availability to bind with the streptavidin in the HABA colorimetric assay. Over time in air, the cellulose chains can rotate the lower energy biotin to the surface to minimize the free surface energy. The reverse phenomenon was observed when samples after 5 months of storage were re-submerged in water for 24 h and 7 days. Both submersion times resulted in complete disappearance of the surface-available biotin in the HABA assays. Normalized FTIR spectra of the samples before and after water submersion showed that the characteristic biotin peaks had no change in absorbance (Figure S7). This supported the hypothesis that the biotin did not leach away from the fibers when submerged in water, but instead rotated below the surface of the fibers due to the water molecules favorably hydrogen bonding with the cellulose hydroxyl groups. For the diagnostic device application, short contact times (up to one hour) did not affect the surface composition of the cellulose nanofibers.

**Figure 7:**
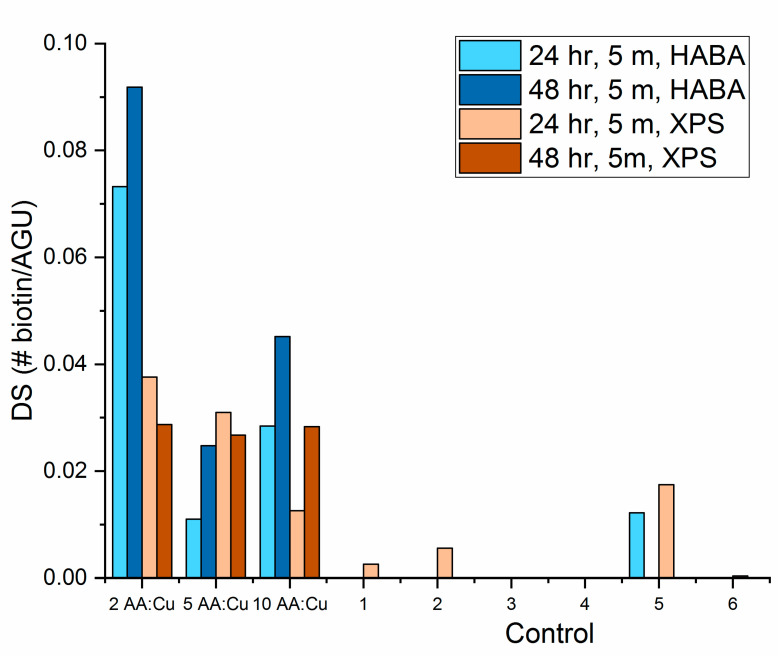
Degree of substitution of biotin on cellulose nanofibers after 5 months of storage calculated from HABA colorimetric assay and XPS survey scans.

The degree of substitution of the biotin after 5 months of storage was also calculated from the XPS results using Equation (3). The ratio of sulfur to carbon was calculated from the XPS survey scans. Figure 7 shows the comparison of the degree of substitution of biotin calculated from HABA and XPS. 

HABA quantified only the amount of biotin available to bind with the streptavidin in solution. The HABA assay did not account for any diffusion limitations, any biotin that was chemically modified, or any biotin that resided in a conformation that made it unavailable to bind with streptavidin. XPS detected all carbon and sulfur present on the sample near the surface. Therefore, it can detect total surface biotin regardless of configuration or chemical modification. Two main disadvantages to XPS for cellulose nanofibers are carbon contamination that can accumulate on the surface of the samples as well as the shadowing effect of the round nanofibers. Both sources of error can reduce the sulfur-to-carbon ratio and resulted in lower degree of substitution (DS) results as seen in the 2 ascorbic acid to copper sulfate (AA:Cu) ratio samples. Overall, the XPS and HABA DS calculations were in agreement.

The degree of substitution of biotin was calculated to be between 0.01 and 0.10 from both the HABA colorimetric assay and XPS. This magnitude of DS translates to one biotin molecule per every 10 to 100 AGU. The repeat unit of cellulose, cellobiose, contained two AGU and had a unit length of approximately 1.03 nm [29]. Therefore, the biotin was, on average, spaced 5 to 50 nm apart. This biotin spacing is useful for filtration and diagnostic device applications as it allows for target and amplification molecules’ immobilization onto the pendant biotin. For example, streptavidin is approximately 5 nm and fits well within the biotin spacing [30]. Amplification molecules rely on proper spacing of the first layer (i.e., biotin) so that subsequent molecules can bind without steric hindrance in sandwich assays.

## 4. Conclusions

Cellulose nanofiber membranes were successfully functionalized with biotin conjugate via click chemistry. The intermediate step of alkyne substitution onto cellulose was dependent on the ratio of solvents, time, and temperature. Both alkyne substitution methods only modified the surface of the nanofibers and did not disturb the bulk. The two-step (RC) compared to the one-step (CA) alkyne substitution method achieved a higher alkyne yield at 80% lower NaOH concentration, less membrane degradation, and six times faster reaction (2 h deacetylation plus 6 h alkyne substitution compared to 48 h alkyne substitution). The two-step method uses milder reaction conditions and is still more efficient than the one-step method, so only the two-step method was used for the click reaction experiments.

The CuAAC click reaction step revealed two major findings:

(1) With all click reaction components, the initial apparent biotin yield was dependent on the ratio of ascorbic acid to copper sulfate, pH, and time.
(2) Click reaction controls confirmed the selectivity and specificity of the reaction; catalysis is possible without a reducing agent, as seen with Control 5 (all click reaction components except ascorbic acid).

Further, the surface-available biotin of the ascorbic acid-free sample (Control 5) was stable with storage over a period of 5 months. The surface-available biotin for the samples with both copper sulfate and ascorbic acid increased with storage. The proposed mechanism of aging is based on the cellulose chain mobility and the minimization of free surface energy. The surface chemistry of the cellulose nanofibers can be controlled by immersing the membrane in water to force the biotin below the surface and storing the membrane in air to allow the biotin to resurface. 

## Figures and Tables

**Table 1:** Click reaction sample components and confocal images of streptavidin-fluorescein-isothiocyanate (FITC) bound to the click reaction sample membranes.

Role		Click Molecule		Catalyst		AA:Cu Ratio	Reaction Time (hours)	Confocal Fluorescent Microscopy
Chemical		Alkyne-RC	Azide-Biotin	CuSO_4_	Ascorbic Acid			
Reaction		X	X	X	X	2, 5, 10	24, 48	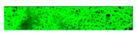
Control	1		X	X	X	-	24	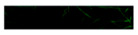
	2		X	X		-	24	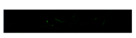
	3	X		X	X	-	24	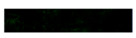
	4	X	X		X	-	24	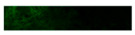
	5	X	X	X		-	24	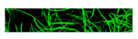
	6	X	X			-	24	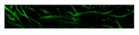

## Supplementary Materials

The following are available online at https://www.mdpi.com/2079-4991/10/6/1172/s1. Figure S1: Fiber diameter distributions from SEM images for as-spun CA, deacetylated CA, and alkyne-RC. Figure S2: Fiber diameter distributions from SEM images of click reaction samples. Figure S3: Raman spectra of regenerated cellulose and alkyne-cellulose nanofibers. Figure S4: XRD plots of (a) one-step (CA) and (b) two-step (RC) during the alkyne substitution reaction. The hydroalcoholic NaOH initially alters the structure of the polymer chains until an equilibrium structure is reached. Figure S5: FTIR spectra of biotin-RC samples for various reaction conditions. Figure S6: Elemental mapping from EDX of biotin-RC fibers. Figure S7: FTIR spectra of biotin-cellulose nanofiber membrane (a) after click reaction, (b) after submerging in water for 1 week, and (c) after performing the HABA assay on (b) sample.
