# Supplementary material for: Biotin-Conjugated Cellulose Nanofibers Prepared via Copper-Catalyzed Alkyne-Azide Cycloaddition (CuAAC) “Click” Chemistry
Source: Nanomaterials (Basel). 2020 Jun 16;10(6):1172. doi: 10.3390/nano10061172 (PMC7353318; doi:10.3390/nano10061172)
Supplement: Supplementary file 1 [file nanomaterials-10-01172-s001.pdf]

Supplementary Materials

Biotin-Conjugated Cellulose Nanofibers Prepared via Copper-Catalyzed Alkyne-Azide Cycloaddition (CuAAC) "Click" Chemistry

Katarina Goodge and Margaret Frey *

Department of Fiber Science and Apparel Design, Cornell University, Ithaca, NY, USA; keg222@cornell.edu
* Correspondence: mfw24@cornell.edu

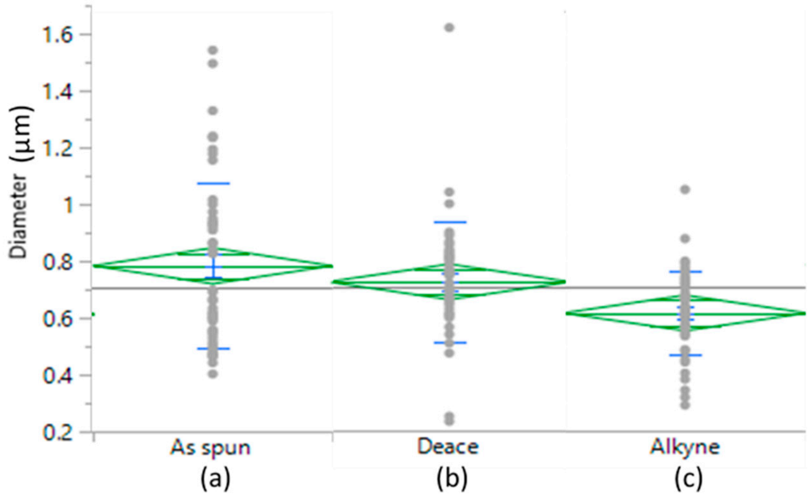

Figure S1. Fiber diameter distributions from SEM images for as-spun CA, deacetylated CA, and alkyne-RC.

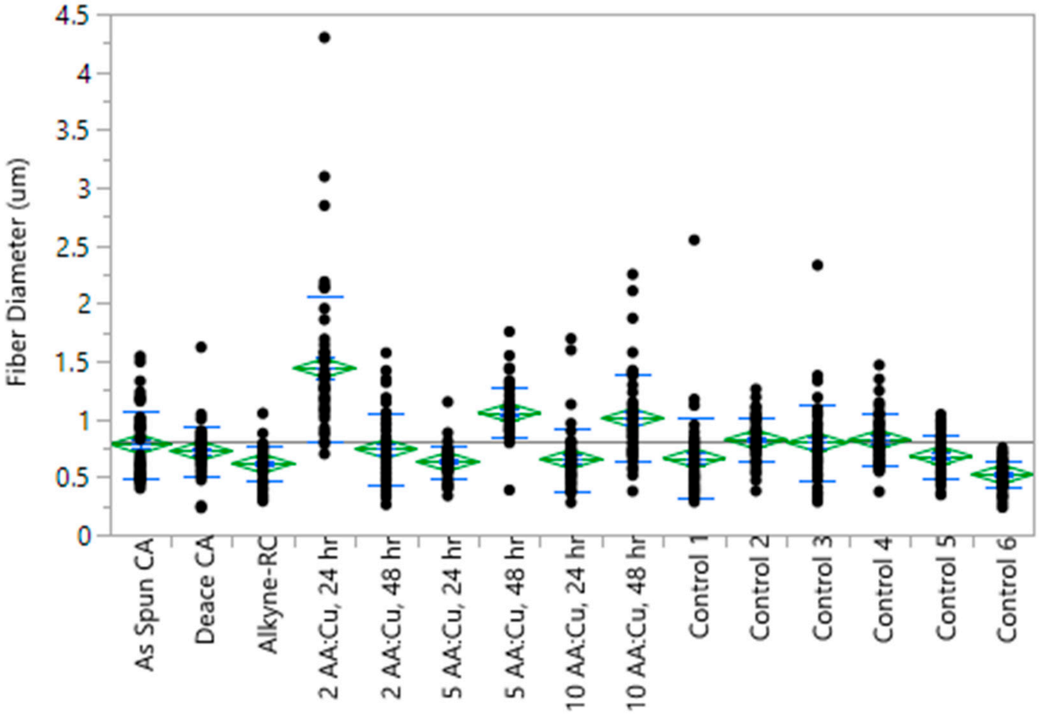

Figure S2. Fiber diameter distributions from SEM images of click reaction samples.

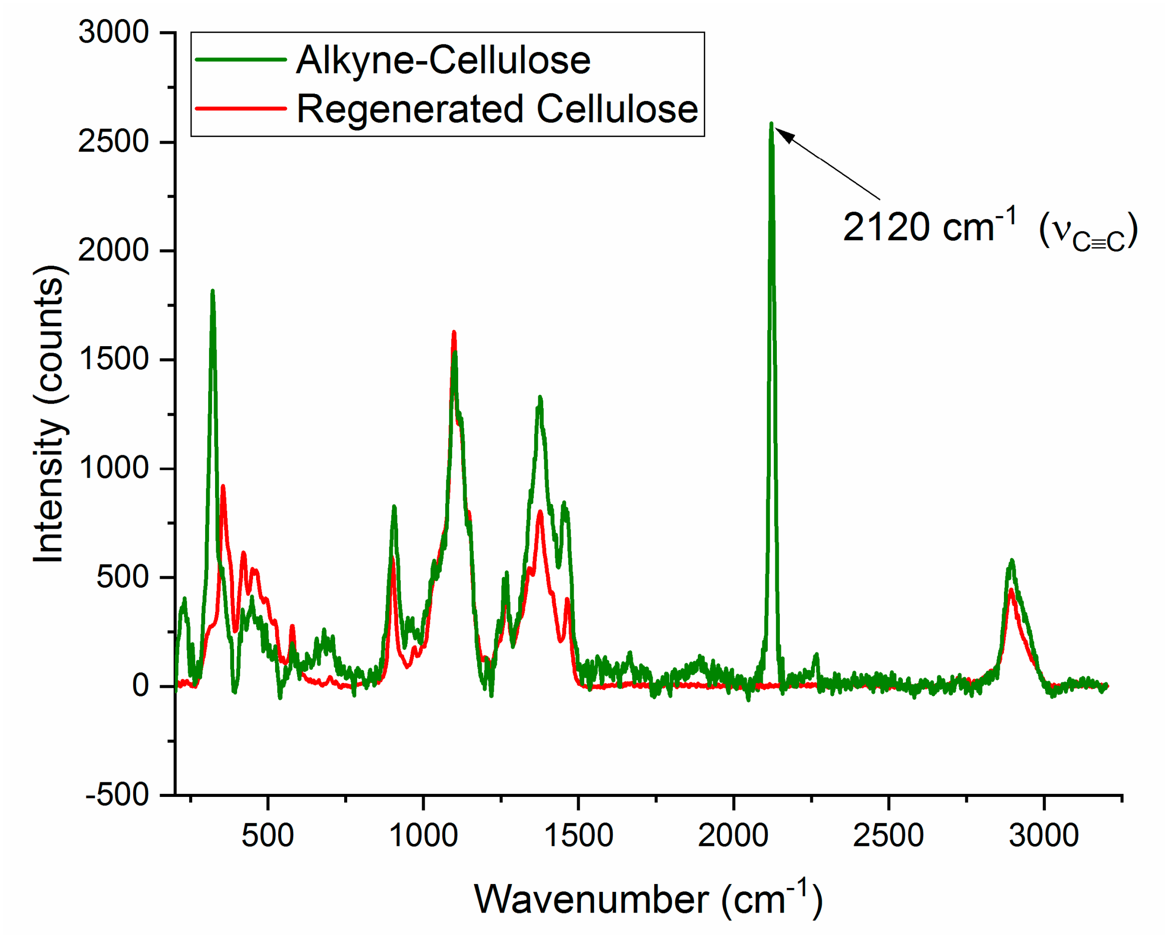

Figure S3. Raman spectra of regenerated cellulose and alkyne-cellulose nanofibers.

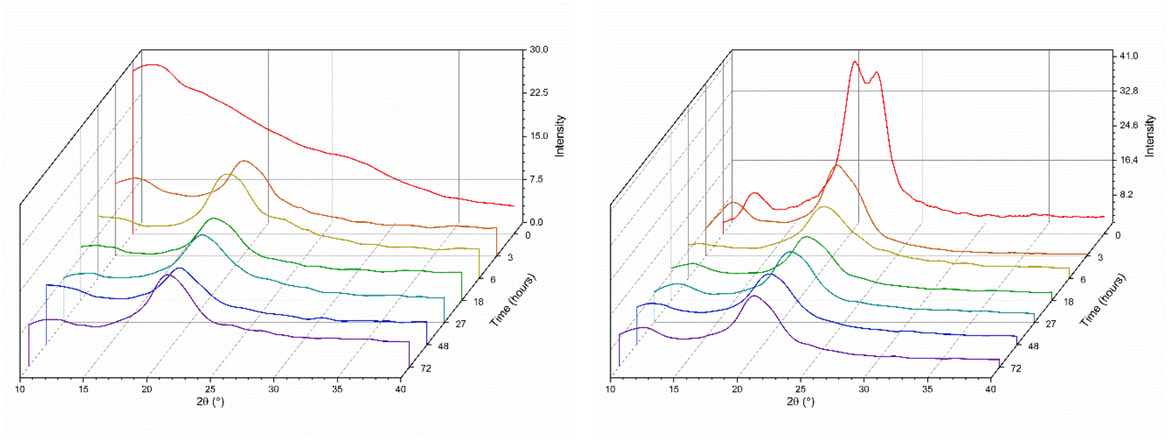

Figure S4. XRD plots of a) one step (CA) and b) two step (RC) during the alkyne substitution reaction. The hydroalcoholic NaOH initially alters the structure of the polymer chains until an equilibrium structure is reached.

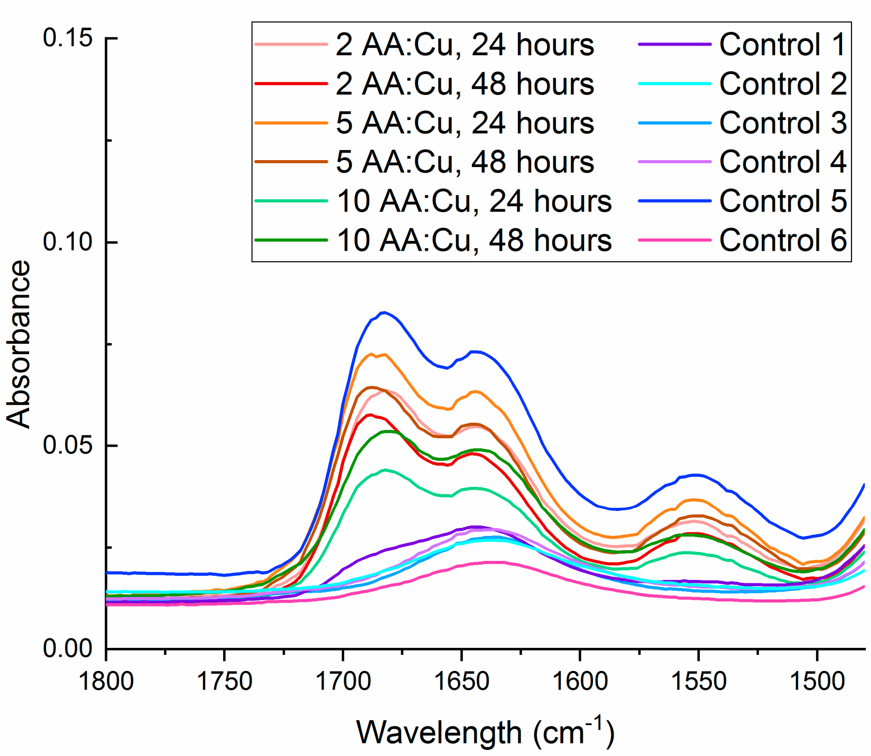

Figure S5. FTIR spectra of biotin-RC samples for various reaction conditions.

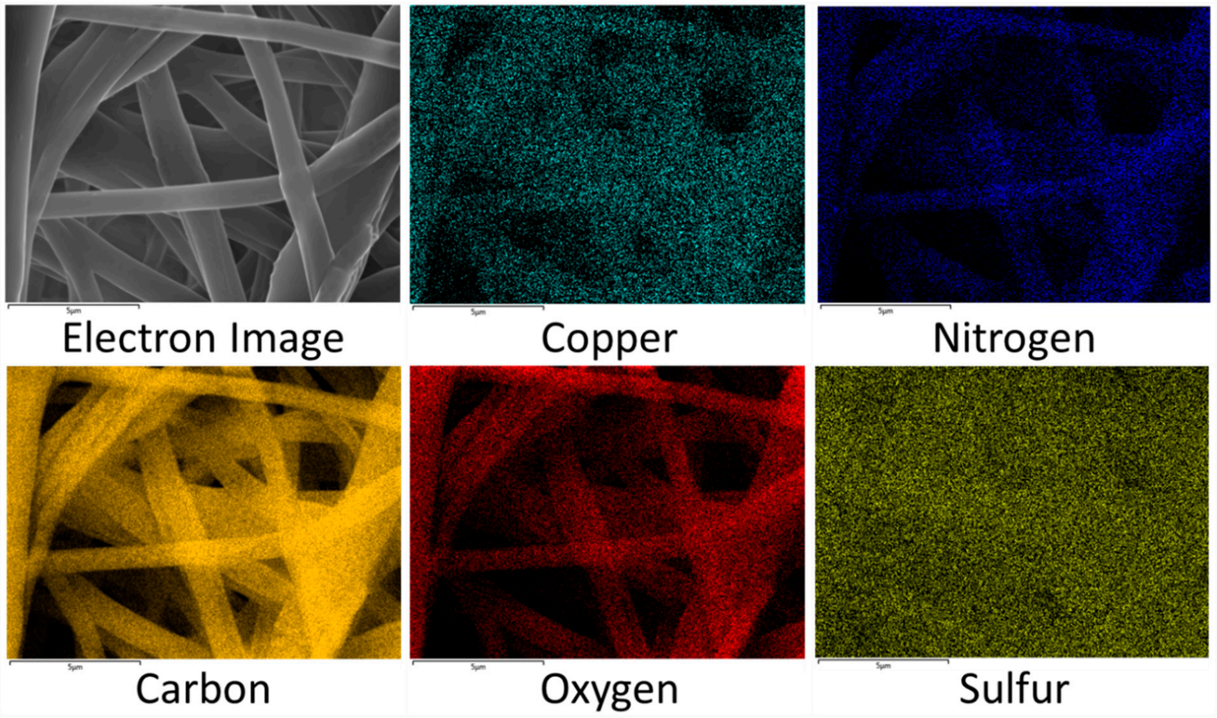

Figure S6. Elemental mapping from EDX of biotin-RC fibers.

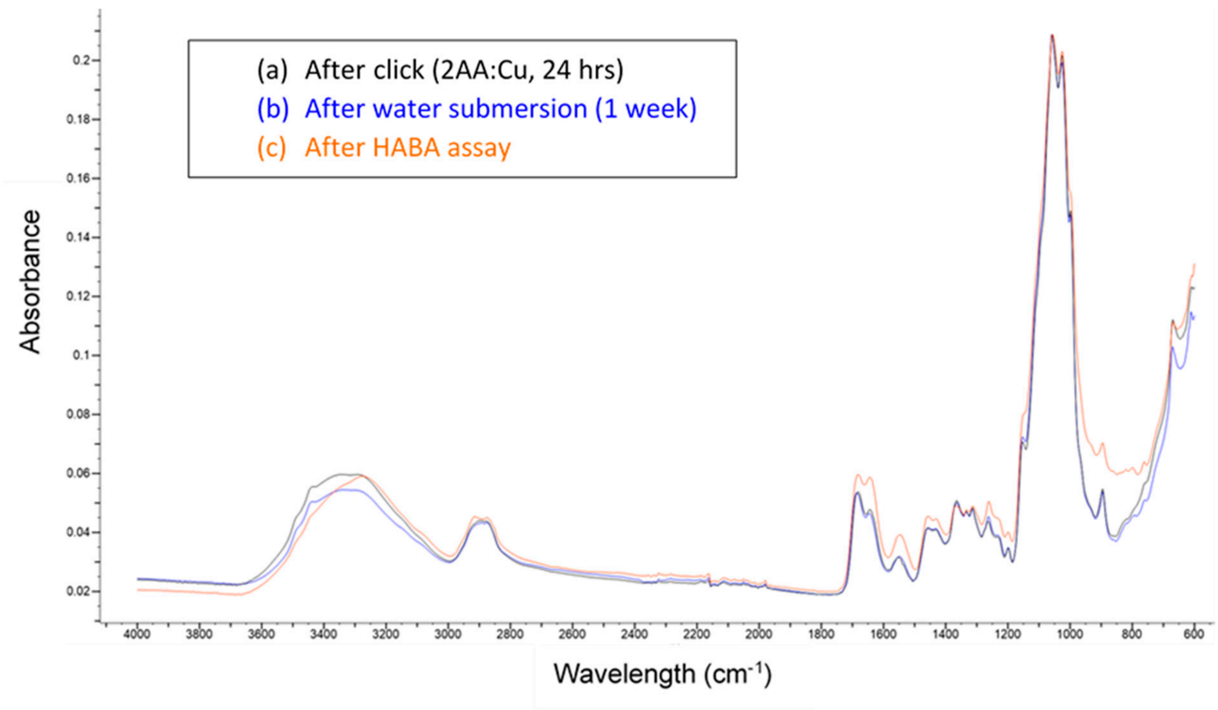

Figure S7. FTIR spectra of biotin-cellulose nanofiber membrane (a) after click reaction, (b) after submerging in water for 1 week, and (c) after performing the HABA assay on (b) sample.
